# Chronic coronary syndrome and new-onset atrial fibrillation or venous thromboembolism: how best to manage antithrombotic therapy strategies

**DOI:** 10.1093/ehjcvp/pvaf021

**Published:** 2025-04-02

**Authors:** Andrea Rubboli, Dominick J Angiolillo, Cecilia Becattini, Gregory Y H Lip

**Affiliations:** Division of Cardiology, Department of Emergency, Internal Medicine and Cardiology, AUSL Romagna, Ospedale S. Maria delle Croci, Ravenna 48121, Italy; Faculty of Medicine, University of Bologna, Campus of Ravenna, Ravenna 48121, Italy; Division of Cardiology, University of Florida College of Medicine, Jacksonville, FL 32209, USA; Internal, Vascular and Emergency Medicine - Stroke Unit, Department of Medicine and Surgery, University of Perugia, Perugia 06123, Italy; Liverpool Centre for Cardiovascular Science at University of Liverpool, Liverpool John Moores University and Liverpool Heart & Chest Hospital, Liverpool L14 3PE, UK

**Keywords:** Chronic coronary syndrome, Atrial fibrillation, Venous thromboembolism, Antithrombotic therapy

## Introduction

Long-term, single antiplatelet therapy (SAPT) with aspirin or clopidogrel is the established antithrombotic therapy (AT) regime for patients with chronic coronary syndrome (CCS).^1^ In subgroups at high ischaemic risk and non-high bleeding risk, dual antiplatelet therapy (DAPT) with aspirin and clopidogrel or ticagrelor or prasugrel, or double AT (DAT) with aspirin and low-dose rivaroxaban are additional options.^1^ Ticagrelor monotherapy is a further AT option which may be considered for patients who have undergone percutaneous coronary intervention (PCI), remain at high ischaemic risk, and were initially treated with ticagrelor-based DAPT.^1^

For CCS patients in whom an indication for oral anticoagulation (OAC), usually with direct oral anticoagulants (DOAC), such as atrial fibrillation (AF) or venous thromboembolism (VTE), arises, no specific recommendations are currently available. Recent data suggest that new-onset AF in patients with CCS is not uncommon (∼1 per 100 patient-years),^2^ whereas the occurrence of VTE may likely be even rarer, although adequate data are lacking. Also lacking are data addressing the AT regime for the two clinical scenarios above, so that the strategies reserved for patients with AF or VTE undergoing PCI, either elective or in the context of acute coronary syndrome, who are in the chronic phase following the index event, i.e. 6 or 12 months later respectively, are generally applied.^1,3,4^ Even for these settings however, the data are limited and are essentially derived from patients with AF who, in the observations where multiple indications for OAC were included, have been reported to account for an average 61% of cases as opposed to an average 10% for patients with VTE.^5^

## Antithrombotic therapy in AF patients with CCS

In AF patients undergoing PCI, OAC monotherapy, preferably with DOAC, is indicated over the long term, after an initial, short (up to 1 week) period of triple AT (TAT) of OAC, aspirin and clopidogrel, followed by a subsequent period of 6–12 months of DAT of OAC and SAPT, preferably with clopidogrel.^1,3^ More prolonged durations of either TAT or DAT may be considered for selected patients at high ischaemic risk, such as those with previous stent thrombosis or undergoing complex PCI or last remaining vessel stenting, and non-high bleeding risk.^1,3^

The long-term OAC monotherapy regime is supported by the three recent randomized clinical trials, OAC-ALONE,^6^ AFIRE,^7^ and EPIC-CAD,^8^ all of which have been conducted in Asian countries, where OAC alone was compared to OAC plus SAPT in AF patients with CCS (*Table 1*). Whilst acknowledging the differences in study design, definition of CCS, and outcomes evaluated in the three trials (*Table 1*),^6–8^ an overall advantage on either the net clinical benefit (i.e. combined occurrence of efficacy and safety outcomes) or safety was consistently reported with OAC monotherapy, mostly with DOAC, with no penalty as regards efficacy.^6–8^ Of note, the weakest benefit was observed in the OAC-ALONE trial,^6^ where the premature termination and the largely prevalent use of vitamin K antagonists (VKA) may have played a role. Also, in the OAC-ALONE trial,^6^ unapproved doses of DOAC were used in ∼20% of cases. And DOAC underdosing in AF patients submitted to PCI has been reported to increase the risk of ischaemic stroke.^9^ In the AFIRE^7^ and EPIC-CAD^8^ trials where the DOAC rivaroxaban and edoxaban respectively, were used in all cases, the superior net clinical benefit of OAC monotherapy was evident (*Table 1*). Whilst in the EPIC-CAD trial,^8^ edoxaban was used at the approved dose, also for Asian populations, of 60 mg once daily (OD), to be reduced to 30 mg OD in the presence of creatinine clearance (CrCl) <50 mL/min or body weight <60 kg or concomitant P-glycoprotein inhibitor treatment, in the AFIRE trial^7^ the doses of rivaroxaban were the ones tested in the J-ROCKET AF trial,^10^ and approved for Japanese patients,^11^ i.e. 15 mg OD, to be reduced to 10 mg OD when CrCl was <50 mL/min, instead of the 20/15 mg OD regime tested in the international ROCKET AF trial.^12^ Indeed, ethnic differences in the risks of thrombosis and bleeding between Asian and white European populations are known, and the so-called East Asian paradox in relation to AT has been well recognized.^13^ Of note, in a pharmacokinetic model in Japanese patients, the rivaroxaban plasma levels with the 15/10 mg and 20/15 mg dosing regimes were comparable.^14^

**Table 1:** Randomized trials comparing OAC monotherapy vs. OAC plus SAPT in AF patients with CCS

	OAC-ALONE^6^	AFIRE^7^	EPIC-CAD^8^
Study design	Prospective, multicentre, open-label, noninferiority	Prospective, multicentre, open-label, noninferiority	Prospective, multicentre, open-label, superiority
No. of pts.	696	2236	1040
Definition of CCS	PCI >1 year earlier	PCI/CABG >1 year earlier or angiographically confirmed CAD not requiring revascularization	PCI/CABG >6 months earlier or acute coronary syndrome treated with PCI/CABG >12 months earlier or significant coronary artery stenosis at coronary angiography/CT scan treated medically
Type of OAC	VKA (75%) DOAC (apixaban, dabigatran, edoxaban, rivaroxaban) (25%)	DOAC (rivaroxaban) (100%)	DOAC (edoxaban) (100%)
Type of SAPT	Aspirin (86%), clopidogrel (24%)	Aspirin (70%), clopidogrel (24%), other P2Y12-inhibitors (4%)	Aspirin (62%), clopidogrel (38%)
Follow-up duration	2.5 years (median)	2 years (median)	1 year
Risk of main clinical outcomes [HR (95% CI)]			
Efficacy	1.16 (0.79–1.72)^a^	0.72 (0.55–0.95)^b^,*	1.23 (0.48–3.10)^c,g^
Safety	0.73 (0.44–1.20)^d^	0.59 (0.39–0.89)^d^,**	0.32 (0.14–0.73)^d,g^
Net clinical benefit	0.99 (0.71–1.39)^e^,***	0.62 (0.47–0.82)^e,g^	0.44 (0.30–0.65)^f^,****

OAC, oral anticoagulation; AF, atrial fibrillation; CCS, chronic coronary syndrome; PCI, percutaneous coronary intervention; CABG, coronary artery bypass grafting; CAD, coronary artery disease; VKA, vitamin K-antagonist; SAPT, single antiplatelet therapy; HR, hazard ratio; CI, confidence intervals; CV, cardiovascular; MI, myocardial infarction; SE, systemic embolism; ISTH, International Society of Thrombosis and Haemostasis; CRNM, clinically relevant nonmajor.

*P < 0.001 (noninferiority); **P = 0.01 (superiority); ***P = 0.016 (noninferiority); ****P < 0.001 (superiority).

a Composite of all-cause death, MI, stroke, or SE.

b Composite of stroke, SE, MI, unstable angina requiring revascularization or all-cause death.

c Composite of all-cause death, MI, ischaemic stroke, or SE.

d ISTH major bleeding.

e Composite of all-cause death, MI, stroke, SE, or ISTH major bleeding.

f Composite of all-cause death, MI, stroke, SE, unplanned urgent revascularization, or ISTH major bleeding or CRNM bleeding.

g Secondary endpoint not undergoing statistical analysis for evaluation of treatment effect.

In summary, in AF patients who are in the chronic phase after PCI, OAC monotherapy, preferably with DOAC, is the recommended long-term AT regime. DOAC should be given at the doses used for stroke prevention in international AF trials, with the possible exception of rivaroxaban which may be given at the reduced dose of 15/10 mg OD in patients in whom concerns on bleeding risk are prevailing^1,3^ or in Asian populations.

## Antithrombotic therapy in VTE patients with CCS

As regards patients with recent or previous VTE undergoing PCI, no dedicated data are available. The limited evidence is obtained from mixed populations where VTE, along with other indications for OAC, is included.^5,15,16^ The main results observed in the available datasets, albeit with no dedicated analysis of VTE patients subgroups and with VKA only as the OAC component of TAT, is that this latter AT regime is associated with an increased risk of bleeding.^5,15,16^ Based on this, as well as on extrapolation from the much larger evidence derived from AF patients, an expert consensus guidance for the management of the AT in patients with recent or previous VTE who are submitted to PCI has been developed.^4^

To start, consideration should be given to whether or not the recommended period of OAC, which at variance of AF may not be indefinite, has been completed.^4^ At least 3 months of OAC, preferably with DOAC, are required when VTE is provoked by a major transient or reversible risk factor, such as, trauma or surgery, whereas indefinite OAC needs to be considered when VTE is unprovoked and/or recurrent.^17^ In comparison to warfarin, all the four DOAC dabigatran, apixaban, edoxaban, and rivaroxaban have consistently shown comparable efficacy and superior safety.^17^ Therapeutic doses of DOAC as tested in VTE clinical trials should be used throughout the entire treatment with the exception of rivaroxaban and apixaban, which can be used at the reduced dose of 10 mg OD and 2.5 mg twice daily (BID) respectively, after the first 6 months of full-dose treatment.^17^

Therefore, in VTE patients undergoing PCI, if at least 3 months of OAC have passed and there is no indication for indefinite OAC, this latter can be interrupted and DAPT with aspirin and clopidogrel or ticagrelor or prasugrel instituted for the following 6–12 months (unless the bleeding risk is deemed high thereby warranting shorter DAPT durations), and followed by SAPT with aspirin or clopidogrel lifelong.^4^ Of note, extended SAPT with aspirin following the standard period of OAC, albeit less effective than OAC for the prevention of VTE recurrence, is nonetheless capable to reduce subsequent episodes compared to no treatment.^17^ If at least 3 months of OAC have not passed and/or there is indication for indefinite OAC, DAPT with aspirin and clopidogrel should be added, thus instituting TAT, which generally needs to be continued for a short period (up to 1 week) and then followed by DAT with OAC and SAPT, preferably with clopidogrel, until the recommended period of OAC is completed or up to 6–12 months when OAC needs to be continued indefinitely.^4^ Upon interruption of DAT, either SAPT, with aspirin or clopidogrel, or OAC alone should be continued long-term depending on whether or not the indication for OAC is indefinite.^4^ Consideration to prolong DAT over the long term may likely be given to selected patients at high ischaemic risk, such as those with previous stent thrombosis, or undergoing complex PCI, or last remaining vessel stenting, and non-high bleeding risk. Whereas the reduced doses of rivaroxaban (10 mg OD) and apixaban (2.5 mg BID) are an option in VTE patients in whom there is an indication for indefinite OAC and the first 6 months of full-dose treatment have been completed,^17^ it is currently untested whether such low doses, whilst effective in preventing recurrent VTE are effective as well in preventing recurrent coronary events. In a historical, randomized, multicentre trial where warfarin alone was compared to aspirin alone for the secondary prevention after myocardial infarction, the superior efficacy observed with warfarin was obtained at a higher than conventional intensity of OAC, namely at a target International Normalized Ratio (INR) of 2.8–4.2.^18^ In the AFIRE^7^ and EPIC-CAD^8^ trials, where OAC monotherapy with the DOAC rivaroxaban and edoxaban respectively, was effective in preventing recurrent coronary events, the DOAC doses were standard, at least for those Asian populations. No relevant evidence is available in support of the efficacy of DOAC doses lower than standard for AF, and even more VTE, for the prevention of coronary events in patients with coronary artery disease. However, in the large population of patients with CCS included in the COMPASS trial,^19^ rivaroxaban monotherapy at the low dose of 5 mg BID, and therefore of 10 mg on a daily basis, was as effective as aspirin in preventing recurrent cardiovascular events.

In summary, in VTE patients who are in the chronic phase after PCI, either SAPT, with aspirin or clopidogrel, or OAC monotherapy, preferably with a DOAC, depending on whether or not the indication for OAC is indefinite, are the recommended long-term AT regimes. The doses of DOAC should be the standard tested in VTE clinical trials with a possible exception for rivaroxaban for which the reduced dose of 10 mg may be considered based on direct evidence in the context of VTE^17^ and indirect evidence in the context of CCS.^19^ In the absence of such data for apixaban 2.5 mg BID, the addition of low-dose aspirin should be considered for indefinite treatment when the reduced dose of apixaban is planned to be continued.

## Antithrombotic therapy in CCS patients with new-onset AF or VTE

Based on the above considerations, as well as on the indirect evidence derived from the opposite scenario, i.e. patients with AF or VTE who are in the chronic phase following PCI, the approach to AT management for CCS patients with new-onset indication for OAC, in particular DOAC, can be summarized as follows.

**Figure 1:**
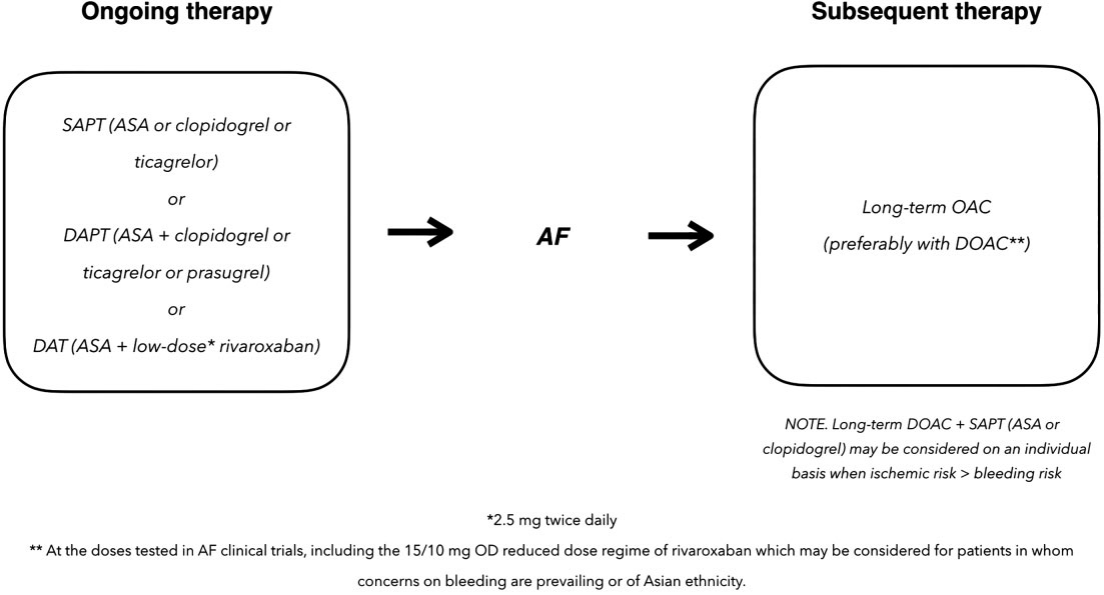
Antithrombotic therapy for CCS patients with new-onset AF. CCS, chronic coronary syndrome; AF, atrial fibrillation; OD, once daily; SAPT, single antiplatelet therapy; DAPT, dual antiplatelet therapy; DAT, double antithrombotic therapy; OAC, oral anticoagulation; DOAC, direct oral anticoagulant.

Irrespective of the ongoing AT, in CCS patients developing AF switching to OAC monotherapy, preferably with a DOAC, is generally warranted in the long term (*Figure 1*). The doses of DOAC should be those tested in AF clinical trials, including the 15/10 mg OD reduced dose regime of rivaroxaban, which may be considered for patients in whom concerns on bleeding are prevailing^1,3^ or of Asian ethnicity.

**Figure 2:**
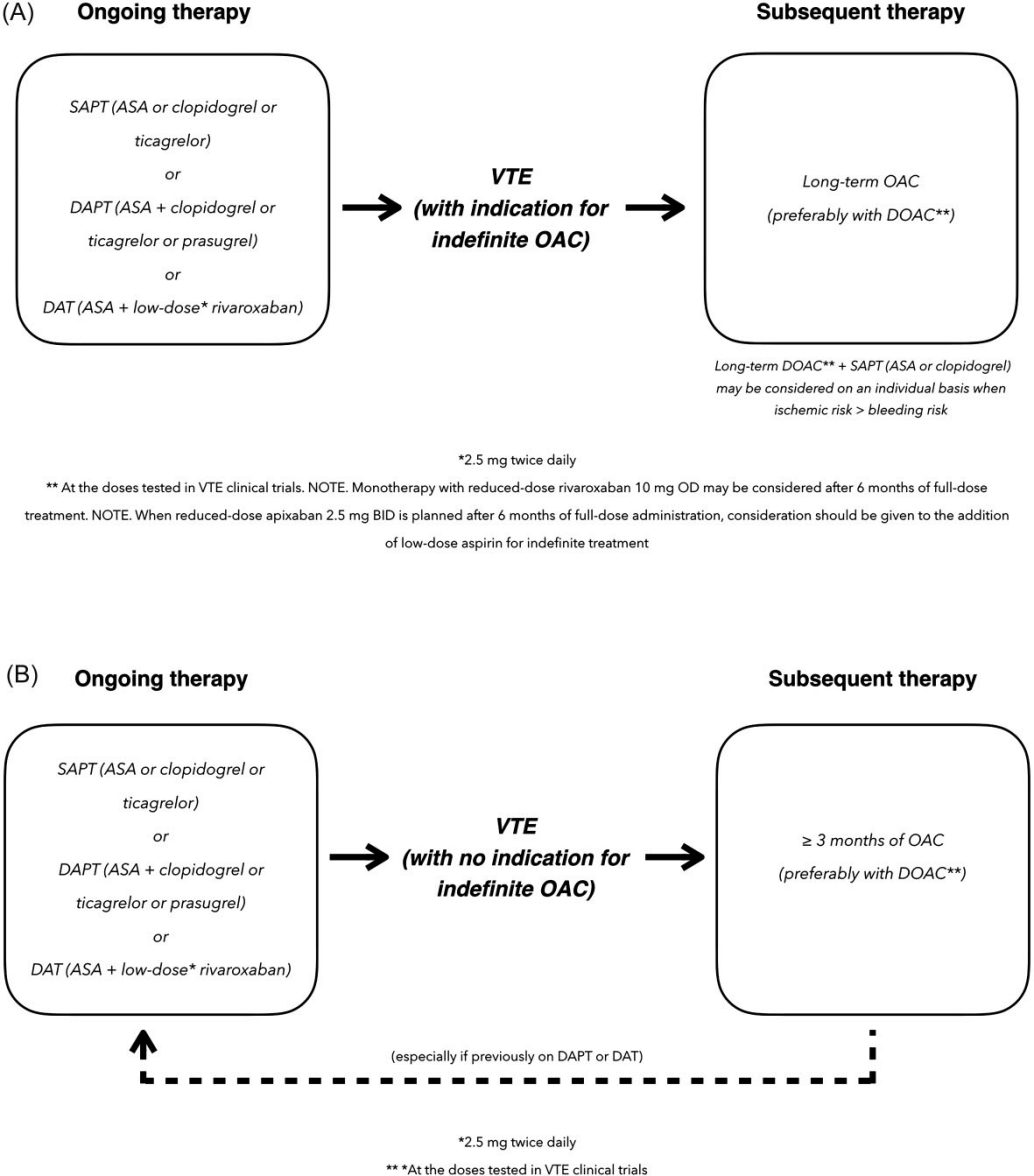
Antithrombotic therapy for CCS patients and new-onset VTE with indication (A) or not (B) for indefinite OAC. CCS, chronic coronary syndrome; VTE, venous thromboembolism; SAPT, single antiplatelet therapy; DAPT, dual antiplatelet therapy; DAT, double antithrombotic therapy; OAC, oral anticoagulation; DOAC, direct oral anticoagulant.

In CCS patients developing VTE, OAC monotherapy, again preferably with a DOAC, is generally warranted, irrespective of the ongoing AT (*Figure 2*A and B). The duration of OAC monotherapy should be guided by whether or not the indication for OAC is indefinite. If yes, OAC needs to be maintained long-term (*Figure 2*A). If not, switching back to the initial AT should be considered, especially for patients who were previously on long-term DAPT or DAT (with ASA and low-dose rivaroxaban) because of the very high ischaemic risk (*Figure 2*B). When the indication for OAC is indefinite and the agent chosen is a DOAC, the dose tested in clinical trials should be used. A possible exception can be made for rivaroxaban, which after the first 6 months of full-dose treatment can be considered for dose reduction to 10 mg OD. When reduction of the apixaban dose to 2.5 mg BID is planned after the first 6 months of full-dose administration, consideration should be given to the addition of low-dose aspirin for indefinite treatment.

## Notes

**Conflict of interest:** A.R. declares that he has received consulting fees or honoraria from AstraZeneca, Bayer, Boehringer Ingelheim, Daichi Sankyo, and Pfizer/Bristol-Myers Squibb. D.J.A. declares that he has received consulting fees or honoraria from Abbott, Amgen, Anthos, AstraZeneca, Bayer, Biosensors, Boehringer Ingelheim, Bristol-Myers Squibb, Chiesi, CSL Behring, Daiichi-Sankyo, Eli Lilly, Faraday, Haemonetics, Janssen, Merck, Novartis, Novo Nordisk, PhaseBio, PLx Pharma, Pfizer, and Sanofi. Also, D.J.A. declares that his institution has received research grants from Amgen, AstraZeneca, Bayer, Biosensors, CeloNova, CSL Behring, Daiichi-Sankyo, Eisai, Eli Lilly, Gilead, Idorsia, Janssen, Matsutani Chemical Industry Co., Merck, Novartis, and the Scott R. MacKenzie Foundation. C.B. declares that she has received consulting fees and honoraria from Bayer, Pfizer/Bristol-Myers Squibb, and Daiichi Sankyo. G.Y.H.L. declares that he has received consulting fees and honoraria from Pfizer/Bristol-Myers, Boehringer Ingelheim, Daiichi Sankyo, and Anthos.

## Data availability

The data underlying this article are available in the article.
